# An assessment of oral cancer knowledge, attitudes, and practices among undergraduate students in Malaysian dental schools

**DOI:** 10.1186/s12903-023-03354-8

**Published:** 2023-08-31

**Authors:** Zheng-Wen Chan, Yi-Fan Phuan, Pei-Yun Ooi, Nuruljannah Nor Azmi, Deepak G.S. Pateel, Hui-Yeng Y. Yap, Shilpa Gunjal

**Affiliations:** 1 Faculty of Dentistry, MAHSA University, Jenjarom, Selangor Malaysia; 2 School of Dentistry, International Medical University, Bukit Jalil, Kuala Lumpur, 57000 Malaysia

**Keywords:** Oral cancer, Dental undergraduates, Knowledge, Attitude, Practices

## Abstract

**Background:**

Oral cancer is a significant public health concern worldwide. Early detection and prevention are crucial in reducing the morbidity and mortality rates associated with this disease. As future dental professionals, dental undergraduates play a vital role in promoting oral health and identifying potential oral cancer cases.

**Methods:**

This study aimed to evaluate the level of oral cancer awareness, knowledge, attitudes, and practices among dental undergraduates in Malaysia. A total of 595 students from years 3, 4, and 5 in both public and private universities participated.

**Results:**

The results showed that a higher percentage of dental undergraduates from private universities were aware of oral cancer and had satisfactory knowledge compared to those from public universities (p < 0.05). Moreover, 59.4% of respondents felt less confident in diagnosing oral cancer during routine dental practice, and 96.1% agreed on the need to increase public awareness of oral health. Interestingly, students from private universities exhibited higher levels of awareness and knowledge regarding oral cancer than those from public universities.

**Conclusions:**

To enhance oral cancer detection and prevention, it is essential to reinforce the current curriculum and provide training to improve diagnostic skills for every dental undergraduate. This will ensure that they are well-equipped with the necessary knowledge and competence to detect and prevent oral cancer effectively.

## Background

Oral cancer is defined as malignant neoplasms in the oral cavity, comprising of buccal mucosa, the anterior two-third of the tongue, the lip, palate, vestibule, alveolus, floor of the mouth, and gingivae [1, 2]. It is otherwise known as oral squamous cell carcinoma (OSCC), as 90% of cancers are originated from squamous cells [3]. Oral cancer is a growing public health concern worldwide. It is a debilitating disease that could severely cause traumatic impacts on a patient’s emotional, functional, and financial aspects, simultaneously reducing their quality of life. Globally, oral cancer is the 13th most common cancer, and it is estimated that there were 377,713 new cases and 177,757 deaths from cancers of the lip and oral cavity in 2020. Oral cancer is more common in men and older individuals, and it is curable if detected at an early stage. The average age of those diagnosed with oral cancer is 63 [4, 5]. It is more common in developing countries than developed countries and is particularly more prevalent in South and South-East Asia. The incidence varies in different parts of the world primarily because of the associated habits for cancer to occur [6, 7].

In Malaysia, oral cancer is ranked as the 19th most common cancer [8]. According to the Malaysian National Cancer Registry, oral cancer is ranked 8th and 4th most common malignancy among Indian males and females [9]. Oral potentially malignant disorders (OPMDs) are abnormal lesions in the oral cavity that have the potential to develop into oral cancer (OSCC). The risk of progression to cancer varies depending on the type of OPMD, but it is always a possibility. The site where OPMDs develop is also dependent on the risk factors involved. The buccal mucosa of betel quid chewers is constantly exposed to micro-abrasions and irritation, which may lead to the development of exophytic growth of the gingiva, buccal sulcus, and buccal mucosa. On the contrary, lesions are typically endophytic and more commonly observed on the lateral border of the tongue and floor of the mouth in smokers and drinkers [10].

Oral cancer is a complex and multifactorial disease. Patients must be exposed to causative factors for a prolonged duration in their life to develop cancer. It was reported that 75% of oral cancers are related to lifestyle choices [11, 12]. Common risk factors include tobacco smoking, alcohol abuse, chewing areca/betel nut preparations (such as Gutkha, Supari, Pan masala, Khaini, and Tambaku) [13], excessive UV radiation, exposure to metal dust or chemicals such as peroxyacetic acid, vitamin, and mineral deficiencies like severe iron deficiency such as in Plummer-Vinson syndrome and immune deficiencies, and association with human papillomavirus type 16.

Dentists have a unique opportunity to play a vital role in the prevention of oral cancer. The oral cavity is easily accessible, making it ideal for opportunistic screenings. During these screenings, dentists should be vigilant for suspicious lesions and perform a risk assessment for any persistent lesions. If any red flags are present, dentists should make appropriate referrals for accurate diagnosis. It is also important for dentists to conduct a thorough history taking for each patient. This will help to identify any risk factors for oral cancer, such as smoking, alcohol use, or a family history of the disease. Dentists should also encourage patients to quit smoking and reduce their alcohol intake. It is crucial for dentists to be equipped with sufficient and up-to-date knowledge and right attitude towards oral cancer as these factors are essential as they determine the right practice for a dental practitioner towards oral cancer [14]. To our knowledge, there have been few studies conducted in Malaysia specifically focusing on the assessment of oral cancer awareness among dental undergraduates across institutions. Therefore, the objective of this comparative study is to assess the level of oral cancer awareness and knowledge among all clinical dental undergraduate students in Malaysia.

## Methods

### Design of study

A descriptive cross-sectional study was conducted among 3rd, 4th, and 5th year Malaysian dental students from March 2021 to May 2021. The chosen study design allows the assessment of oral cancer awareness among dental undergraduate students in Malaysia as it provides a snapshot of the current level of knowledge and awareness without considering changes over time. The study focused on 3rd, 4th, and 5th year Malaysian dental students who possess clinical knowledge necessary to answer the questionnaire accurately.

Ethical approval for the study (RMC/EC44/2021) was obtained from the Research Management Centre, MAHSA University. All 13 dental schools in Malaysia were included in the present research. During the period of the study, there was a combined enrollment of 2343 students across their 3rd, 4th, and 5th years of academic pursuit. Among them, a count of 971 hailed from government-funded public universities, with the remaining students originating from private institutions. The initial step involved emailing the online questionnaire to dental school Deans to get permission to involve students. Then, students in the study were contacted through their year’s supervising faculty, who helped with communication and data collection. Paper forms were also distributed to peers within and outside the institutions to promote increased participation. At the outset of the study, students were introduced to the research’s purpose through a participant information sheet. Participation in the study was entirely voluntary, and no incentives were provided upon completing the questionnaire. To ensure confidentiality, students’ personal information was safeguarded, and informed consent was obtained from all participants involved in the study.

### Participant selection criteria and exclusions

The study’s inclusion criteria encompassed dental students in their 3rd, 4th, and 5th years of study, all of whom were enrolled in dental schools situated within Malaysia. Participation in the study was contingent upon the students’ voluntary provision of informed consent and their expressed willingness to engage in the research process.

Conversely, students who chose not to grant consent for their participation were excluded from the study. This exclusion criterion ensured that only participants who were fully agreeable to taking part were involved in the research, maintaining the integrity of the study’s outcomes.

### Study instrument

A pretested questionnaire was distributed to all the students through Google Forms to evaluate their fundamental knowledge and awareness of oral cancer. The validated questionnaire has been obtained from Gunjal et al. with modifications and additional questions [15]. The questionnaire consists of 47 items (one open-ended and 46 close-ended) with five sections namely “Sociodemographic data” (4 questions), “General Awareness” (7 close-ended and one open-ended question), “Knowledge” (22 questions), “Attitude” (7 questions) and “Practice” (6 questions) to assess students’ habits during the oral examination, knowledge on signs and symptoms of oral cancer, risk factors and students’ desire to receive further information on oral cancer. Responses were given as “Yes”, “No”, and “Do not know”.

### Sample size determination

The sample size was calculated using the two proportions formula in the Power and Sample Size Program. The confidence level was set at 95% (alpha, 𝞪 = 0.05), and the power (1-𝛃) was set to 0.8. The proportions used in the calculation were P_o_ = 0.93 and P_1_ = 0.88. Taking into account an expected 10% missing data, the minimum sample size required for the study was determined to be 593.

### Statistical analysis

Data collected was entered, cleaned, coded, and analyzed with IBM SPSS Statistics Version 26 for Windows. Descriptive statistics were used to determine the demographic characteristics. The results were then analyzed using the Pearson chi-square test to determine the difference in general awareness and knowledge regarding oral cancer between public and private dental schools in Malaysia. Multiple linear regression was used to assess the predictors associated with oral cancer knowledge. Model assumptions were met, no multicollinearity problem detected and interaction term was found to be non-significant. *P-*value less than 0.05 would be considered statistically significant.

## Results

### Sociodemographic characteristics

A total of 595 participants responded to the questionnaire, of which 256 (43.0%) [UM 73(12.3%), USM 32(5.4%), UKM 35(5.9%), UITM 29(4.9%), IIUM 42(7.1%), USIM 45(7.6%)] from public and 339 (57.0%) [AIMST 57(9.6%), PIDC 35(5.9%), MAHSA 108(18.2), IMU 31(5.2%), MMMC 33(5.5), SEGI 46(7.7%), LINCOLN 29(4.9%)] were from private universities. Respondents from the third, fourth, and final years included 22.2% (132), 36.1% (215), and 41.7% (248) respectively. Most of the respondents’ age ranged from 22–24 years [77.1% (459)], followed by ≥ 25 years [14.5% (86)] and ≤ 21 years [8.4% (50)]. About 56% (333) of the surveyed students were females and 44% (262) were males.

### Awareness and knowledge of oral cancer

As the study aimed to assess and compare the level of knowledge, attitude, and practices of oral cancer among the participants, the responses “No” and “Do not know” were combined into “No” for data analysis using a chi-square test. Overall, the difference in general awareness and knowledge regarding oral cancer were statistically significant between dental students from public and private universities (p < 0.05) (Table 1).

**Table 1:** Overall association between public and private universities regarding awareness, knowledge, attitude, and practice

Characteristics		Frequency (%)		X^2^(df)	p-value
		Public School	Private School		
Awareness	Inadequate	27 (10.5)	18 (5.3)	5.722 (1)	0.017
	Adequate	229 (89.5)	321 (94.7)		
Knowledge	Inadequate	40 (15.6)	32 (9.4)	5.246 (1)	0.022
	Adequate	216 (84.4)	307 (90.6)		
Attitude	Inadequate	66 (25.8)	83 (24.5)	0.131 (1)	0.718
	Adequate	190 (74.2)	256 (75.5)		
Practice	Inadequate	22 (8.6)	27 (8.0)	0.076 (1)	0.782
	Adequate	234 (91.4)	312 (92.0)		

Chi-square test, p < 0.05 (Statistically significant)

A more significant proportion of dental undergraduates from private universities (94.7%) were aware of oral cancer than public universities (89.5%). Based on a multiple linear regression analysis, for every additional one point in the score, the mean awareness score decreased among dental students from public universities in comparison to private universities when adjusted for age and gender [b (95% CI) = 0.029 (0.008 to 0.051), p = 0.008]. (Table 2) Most participants (~ 90%) agreed that oral cancer was chiefly preventable and had the best chance of a successful cure when diagnosed early (Table 3). The respondents have given various ways of detecting oral cancer including clinical or self-examination, history taking, biopsy, radiographs, computed tomography scans, magnetic resonance imaging, staining method, VELscope, ViziLite Oral Cancer Screening, tumor markers, and Human Papillomavirus (HPV) testing.

**Table 2:** Predictors associated awareness towards oral cancer by linear regression.

Variable	Simple linear regression (univariable analysis)			Multiple linear regression (multivariable analysis)		
	Unadjusted b	95% CI	P-value	Adjusted b	95% CI	P-value
Age	0.017	-0.006 to 0.040	0.145	-	-	-
Gender	0.023	0.001 to 0.045	0.037	-	-	-
Public Vs Private	0.035	0.013 to 0.057	0.002	0.029	0.008 to 0.051	0.008
Year	0.038	0.024 to 0.052	< 0.001	0.036	0.022 to 0.050	< 0.001

b, Regression coefficient

**Table 3:** General awareness, attitude, and practice of the respondents towards oral cancer.

Questions	Public, n (%)		Private, n (%)		Overall, n (%)		P-value
	No	Yes	No	Yes	No	Yes	
**GENERAL AWARENESS**							
Have you heard of oral cancer?	1 (0.4)	255 (99.6)	2 (0.6)	337 (99.4)	3 (0.5)	592 (99.5)	0.734
Is oral cancer preventable?	34 (13.3)	222 (86.7)	31 (9.1)	308 (90.9)	65 (10.9)	530 (89.1)	0.109
Is oral cancer treatable?	36 (14.1)	220 (85.9)	20 (5.9)	319 (94.1)	56 (9.4)	539 (90.6)	0.001
Is oral cancer contagious?	211 (82.4)	45 (17.6)	280 (82.6)	59 (17.4)	491 (82.5)	104 (17.5)	0.956
Does the risk of getting oral cancer increase with age?	31 (12.1)	225 (87.9)	49 (14.5)	290 (85.5)	80 (13.4)	515 (86.6)	0.406
Does oral cancer spread to other parts of the body?	34 (13.3)	222 (86.7)	29 (8.6)	310 (91.4)	63 (10.6)	532 (89.4)	0.064
Do you know the various ways of detecting oral cancer?	74 (28.9)	182 (71.1)	79 (23.4)	259 (76.6)	153 (25.7)	441 (74.1)	0.127
**GENERAL ATTITUDE**							
Do you know anyone who has oral cancer?	180 (70.3)	76 (29.7)	260 (76.7)	79 (23.3)	440 (74.0)	155 (26.0)	0.079
Do you think you are competent to detect oral cancer?	162 (63.3)	94 (36.7)	191 (56.3)	148 (43.7)	353 (59.3)	242 (40.7)	0.088
Will you deny treatment to patients with oral cancer?	231 (90.2)	25 (9.8)	300 (88.5)	39 (11.5)	531 (89.2)	64 (10.8)	0.498
Would you get yourself screened for oral cancer?	32 (12.5)	224 (87.5)	40 (11.8)	299 (88.2)	72 (12.1)	523 (87.9)	0.795
Would you advise your friends and family to go for oral cancer screening routinely?	40 (15.6)	216 (84.4)	39 (11.5)	300 (88.5)	79 (13.3)	516 (86.7)	0.142
Do you feel that oral cancer awareness campaigns are effective?	69 (27.0)	187 (73.0)	91 (26.8)	248 (73.2)	160 (26.9)	435 (73.1)	0.976
Do you think more oral cancer awareness campaigns should be carried out?	12 (4.7)	244 (95.3)	11 (3.2)	328 (96.8)	23 (3.9)	572 (96.1)	0.366
**GENERAL PRACTICE**							
Have you ever informed your patients about the risk factors of oral cancer?	53 (20.7)	203 (79.3)	85 (25.1)	254 (74.9)	138 (23.2)	457 (76.8)	0.211
Have you ever advised patients to avoid the risk factors of oral cancer?	45 (17.6)	211 (82.4)	63 (18.6)	276 (81.4)	108 (18.2)	487 (81.8)	0.753
Do you examine the patient’s oral cavity routinely for signs of oral cancer?	38 (14.8)	218 (85.2)	46 (13.6)	293 (86.4)	84 (14.1)	511 (85.9)	0.658
Do you record tobacco and alcohol use in personal history?	9 (3.5)	247 (96.5)	8 (2.4)	331 (97.6)	17 (2.9)	578 (97.1)	0.402
Do you examine head and neck lymph nodes of suspicious patients?	26 (10.2)	230 (89.8)	30 (8.8)	309 (91.2)	56 (9.4)	539 (90.6)	0.589

The level of knowledge was determined by identifying risk factors and recognizing the signs and symptoms of oral cancer. About 90.6% of the participants from private universities were more informed concerning risk factors and malignant changes for oral cancer against public universities (84.4%) (Table 1). In general, a high percentage of the surveyed students (> 90%) recognized smoking, smokeless tobacco, betel quid chewing, and excessive radiation exposure as the main risk factors contributing to oral cancer development. Interestingly, the majority of the students (87.6%) agreed that HPV had been associated with oral cancer. Among all the risk factors, mouthwash was the least likely to be selected by the respondents (17.3%). By comparison, a considerable percentage of dental students from private universities (80.8%) than public universities (66%) believed that chronic irritation from cheek or lip biting would cause malignant changes to the oral mucosal.

When asked to choose the signs and symptoms of oral cancer, a total of 532 (89.4%) of the participants identified that a non-healing ulcer for more than two weeks is the most common clinical manifestation of oral cancer, followed by the mixture of red and white lesion [479 (80.5%)], swelling [388 (65.21%)], red lesion [381 (64.03%)] and white lesions [363 (61.01%)]. Inspiringly, only a small percentage of participants [11(1.85%)] did not know the various signs and symptoms of oral cancer. A significant number of dental students from public universities [220 (85.94%)] and private universities [287 (84.66%)] stated that the tongue was the most common site for oral cancer.

### Attitude and practice of oral cancer

Generally, the difference in attitude and practice of oral cancer between dental graduates from public and private universities was statistically not significant (p > 0.05) (Table 2). Overall, more than half of the students [353 (59.3%)] from both groups felt less confident about diagnosing oral cancer during routine dental practice (Table 3). Most of them [572 (96.1%)] agreed on the necessity to raise the public’s dental health awareness by conducting more oral cancer awareness campaigns. Encouragingly, a large part of the respondents [531 (89.2%)] claimed that they would not deny treatment to the oral cancer patient. A significant percentage (> 85%) of the surveyed students would get themselves screened for oral cancer and advise their friends and family to go for screening routinely.

As to the practices of the undergraduates, 85.9% reported regular examination of the patient’s oral mucosa for signs of oral cancer, including 90.6% will examine head and neck lymph nodes of suspicious patients (Table 3). Furthermore, most of the participants (97.1%) recognized the importance of tobacco and alcohol use history in identifying high-risk habits of patients. More than 75% of them would inform and advise their patients about the dangers of risk factors regarding oral cancer. Regarding appropriate referral of patients with suspicious lesions, dentists and oral maxillofacial surgeons are the top two choices, with 77.7% and 50%, respectively.

## Discussion

The present study received more significant responses from females when compared to males. This difference is mainly attributed to the fact that a greater number of female students enrolled in dentistry than males and previous studies conducted in Malaysian universities have also depicted similar results. [15–17]. The same trend is observed in other countries as well like Nepal, India, and Brazil [18–20]. Both public and private university dental students possess a good level of awareness regarding oral cancer, with no significant difference between the two groups (p > 0.05).

Cancer survival rates are approximately 80 to 90% when detected at the earliest [21]. More than 90% of patients with Stage I or II diseases survive their first year, and approximately 75% survive for as long as five years [6]. Stage 3 and 4 carcinomas will kill almost half of the patients in two years and as many as 60% in five years. Studies have shown that two out of three oral cancers are diagnosed in advanced stages III or IV when treatment options are limited with a poor prognosis [22, 23]. These data put a great emphasis on the benefit of early diagnosis [24]. From this study, there is a consensus among the students that oral cancer is a preventable disease. However, there is a statistically significant difference in the perception of treatability between the two groups, with a higher percentage of dental students from private universities (94.1%) compared to public universities (85.9%) agreeing that oral cancer is treatable (p < 0.05). It is important to emphasize to dental students, as future practitioners, the significance of early detection, as it enables better treatment outcomes. Increasing their understanding of the importance of early detection can contribute to higher survival rates for patients. Furthermore, efforts can be made to enhance awareness of the available treatment options for oral cancer. Promoting interdisciplinary collaboration and providing continual education and training on oral cancer can further support dental students in their future roles as healthcare professionals.

Smoking, smokeless tobacco, betel quid chewing, and excessive radiation exposure were correctly identified as the risk factors of oral cancer receiving percentages (> 90%) by dental students among both public and private dental schools. On the other hand, the less known risk factors like alcohol consumption, positive family history of oral cancer, and being immunosuppressed or immunocompromised were identified with lower percentages (> 85%). Similar results have been obtained from a study by Soares et al. [18], Rai et al. [20], and Dubai et al. [17] which emphasizes the need for narrowing the gap in knowledge with more student education. It is recommended to place special emphasis on educating students about alcohol consumption, positive family history, and immunosuppression. Providing targeted training and resources can enhance their understanding of these factors and their connection to oral cancer.

In this study, dental students from private dental schools have a higher level of knowledge (90.6%) in comparison to those from public dental schools obtaining percentages of (84.4%) with a difference of (6.2%) and is statistically significant with (p < 0.05). This difference may be attributed to the fact that the total number of dental students in the public dental schools is vastly outnumbered by those from the private schools. Regardless, it is also vital to ensure equal opportunities for education, considering the potential impact of student numbers on knowledge disparities between private and public dental schools. Striving for equal access to education and resources across institutions through collaboration and knowledge sharing promotes a level playing field for all dental students.

The present study also revealed that non-healing ulcers for more than two weeks were identified as the most common clinical manifestation of oral cancer (89.4%). The study by Srivastava et al. also revealed similar results that non-healing ulcers as the most common clinical manifestation (89.9%) [19]. A study by Dubai et al. also showed similar results with ulcer and oral bleeding; both received the same and the greatest number of responses (71.0%) followed by swelling (61.5%) [17]. It is important for dental students to receive focused education on recognizing and evaluating non-healing ulcers, enabling them to conduct thorough examinations and make appropriate referrals when necessary. Additionally, students should also be familiarized with other common signs and symptoms such as oral bleeding and swelling, ensuring they can identify potential cases effectively.

Students from both public and private universities were able to identify the most common site of oral cancer, which is the tongue (85.1%) which is similar to the result reported by Soares et al., in which the tongue was also described as the primary tumour site (53.58%) [18]. Meanwhile, another study obtained contradictory results where the floor of the mouth was considered the most common site of oral cancer than the tongue [25]. While the tongue is typically considered the primary site of oral cancer, students should be aware of alternative perspectives, such as the floor of the mouth. This knowledge will promote a comprehensive and open-minded approach to patient evaluation and diagnostic decision-making. It is important to foster critical thinking and promote evidence-based practice among dental students. Encouraging students to critically evaluate research findings and consider supporting evidence will enable them to make informed clinical judgments and provide optimal care based on the best available evidence.

The findings of the present study provide valuable insights into the knowledge and perceptions of oral cancer among Malaysian dental students. However, it is important to interpret these results with caution due to certain limitations. Firstly, the response rate from public and private universities was slightly unequal, which may introduce some bias. Additionally, the questionnaire study was conducted online because of the impracticability caused by the COVID-19 pandemic, which could have influenced the participants’ responses. Furthermore, the subjective nature of the responses raises the question of whether there were any associated online referrals prior to completing the survey. It is also essential to consider the potential non-response bias that might have affected the study results. Future research should take these aspects into account to further strengthen the validity and generalizability of their findings.

## Conclusions

The present study reveals notable differences in knowledge and awareness levels regarding oral cancer among dental students at private and public universities, with higher levels observed among students from private universities. However, it is encouraging to note that all dental students, regardless of university affiliation, displayed positive attitudes and practices related to oral cancer. To ensure successful preventive approaches, it is crucial to reinforce the current curriculum and provide continued education and training on oral cancer for dental undergraduates. Emphasis should be placed on developing their diagnostic skills, equipping them with the necessary knowledge and competence to effectively prevent, detect, and manage oral cancer cases. By prioritizing ongoing education and training, dental students will be well-prepared to contribute to the prevention and early detection of oral cancer, ultimately improving patient outcomes and reducing the burden of this disease. Continued efforts in enhancing oral cancer education among dental undergraduates will play a vital role in promoting oral health and well-being in the community.

## Data Availability

The data analysed during this present study are available from the corresponding authors on request.
